# Ketogenic diets and physical performance

**DOI:** 10.1186/1743-7075-1-2

**Published:** 2004-08-17

**Authors:** Stephen D Phinney

**Affiliations:** 1 6108 Boothbay Court, Elk Grove, CA 95758, United States of America

## Abstract

Impaired physical performance is a common but not obligate result of a low carbohydrate diet. Lessons from traditional Inuit culture indicate that time for adaptation, optimized sodium and potassium nutriture, and constraint of protein to 15–25 % of daily energy expenditure allow unimpaired endurance performance despite nutritional ketosis.

## Introduction

In the opinion of most physicians and nutrition scientists, carbohydrate must constitute a major component of one's daily energy intake if optimum physical performance is to be maintained [1]. This consensus view is based upon a long list of published studies performed over the last century that links muscle glycogen stores to high intensity exercise. It has also been reinforced by the clinical experience of many physicians, whose patients following low carbohydrate formula or food diets frequently complain of lightheadedness, weakness, and ease of fatigue.

During the time that this consensus view of the necessity of carbohydrate for vigorous exercise was forming, the last pure hunting cultures among the peoples of North America finally lost out in competition with expanding European cultural influences. Between 1850 and 1930, the routine consumption of carbohydrates spread north from the U.S. Plains States through central Canada, where the indigenous peoples had heretofore made at most seasonal use of this nutrient class. However the last of these groups to practice their traditional diet, the Inuit people of the Canadian and Alaskan Arctic regions, were luckily observed by modern scientists before their traditional dietary practices were substantially altered. The reports of these early scientists imply that the Inuit people were physically unhampered despite consuming a diet that was essentially free of identifiable carbohydrate.

Given this juxtaposition of clinical research results favoring carbohydrate against observed functional well-being in traditional cultures consuming none, it is an interesting challenge to understand how these opposing perspectives can be explained. This paper will review the observations of early explorer scientists among the Inuit, track the controversy that they stimulated among nutritionists in the last century, and utilize some of the forgotten lessons from the Inuit culture to explain how well-being and physical performance can be maintained in the absence of significant dietary carbohydrate.

## The origins of carbohydrate supremacy

Until the development of agriculture over last few millennia, our human ancestors' consumption of dietary carbohydrate was opportunistic. As some groups adapted to hunting and fishing for their sustenance, they were able to move into temperate and then arctic regions, where limited access to wild grain, nuts, and fruit dictated sustained dependence upon fat and protein as primary sources of dietary energy.

With the development of agriculture came the ability to grow and store grain, allowing societies to remain in a stable physical location, build permanent dwellings, and potentially stimulating the development of written language (those early stone tablets would have been difficult to transport from camp to camp on a dog sled). Starting from locations in the Middle East and Asia, cultures based upon agricultural wheat and rice spread over 5 millennia to dominate Europe, Africa, and the Americas.

With its ability to support a non-nomadic life style, greater population density, and permanent communities; there were clear advantages of agriculture-based societies over those based upon hunting and fishing, particularly as agricultural communities built the infrastructure to support trade and transport. Given its success in this competition of cultures (and by implication, the competition of their diets), it is an easy assumption that a grain-based diet is functionally superior to one based upon the meat and fish (fat and protein) of the hunting societies that they superseded.

As the science of nutrition developed in the early 20^th ^Century, numerous comparative studies were undertaken to assess differences between diets. Although there were some advocates of low carbohydrate diets (eg, the Banting diet of the 19^th ^Century, promoted for weight loss and diabetes control), the prevailing premise for these studies was that carbohydrate was a necessary nutrient for optimum human health and function. Among studies confirming this view, a classic was the 1939 study by two Danish scientists, Christensen and Hansen [2]. They did a crossover study of low carbohydrate, moderate carbohydrate, and high carbohydrate diets, each lasting one week. At the end of each diet, the subjects' endurance time to exhaustion on a stationary bicycle was assessed. Compared to the mean endurance time on the low carb diet of 81 minutes, the subjects were able to ride for 206 minutes after the high carb diet.

During the Second World War, another oft-cited study was performed, this time examining the practicality of pemmican (a mixture of dried meat and fat) as a light-weight emergency ration for soldiers. This experiment by Kark et al [3] involved abruptly switching soldiers in winter training in the Canadian Arctic from standard carbohydrate-containing rations to pemmican. This study only lasted 3 days, as the soldiers rapidly became unable to complete their assigned tasks, which included pulling loaded sleds 25-miles per day through deep snow.

With the resurgence of biomedical science in the 1960's came development of the percutaneous needle biopsy, facilitating assessment of intra-muscular fuel stores and metabolism. This led to the concept of muscle glycogen as the limiting fuel for high intensity exercise [4] and to the nutritional strategy of carbohydrate loading [5]. The clear consensus that developed from this research was that fat had limited utility as a fuel for vigorous exercise, and that humans are physically impaired if given a low carbohydrate diet.

## The hunter's counterpoint – practical observations on ketogenic diets

Although high-carbohydrate diets might be more effective in short-term tests of high-intensity exercise, there are multiple clues in the published literature that the debilitating effects of ketogenic diets are overstated. Not only is there the demographic evidence that whole populations of people lived for millennia as hunters, but there are many reports of Europeans crossing over to live within the cultures of these hunting societies without apparent impediment.

One of the earliest documented demonstrations of physical stamina during a ketogenic diet was the Schwatka 1878–80 expedition in search of the lost Royal Navy Franklin expedition. The Schwatka expedition, sponsored by the *New York Herald *and the American Geographical Society, departed from the west coast of Hudson's Bay in April of 1879 with 4 Caucasians, 3 families of Inuits, and 3 heavily laden dog sleds. Totaling 18 people, they started out with a month's supply of food (mostly walrus blubber) and a prodigious supply of ammunition for their hunting rifles. After covering over 3000 miles on foot over ice, snow and tundra, all 18 members of the original party plus their 44 dogs returned to Hudson's Bay in March of 1880. Once their initial provisions were depleted, the expedition's only source of additional food was hunting and fishing, as there were no other sources of supply along their route.

The leader of this expedition, Lt. Frederick Schwatka, was a graduate of both West Point and Bellevue Hospital Medical College. His summary of the expedition was published as a news article in the *New York Herald *in the Fall of 1880, but his written diary was lost for 85 years until its discovery and publication by the Marine Historical Association of Mystic CT in 1965 [6]. This fascinating 117-page saga describes how Schwatka, a frontiersman and U.S. Army surgeon, collaborated with his Inuit guides to accomplish a remarkable feat of physical endurance.

In one notation, Schwatka provides an interesting insight into his weaning from their initial supply of carbohydrate-containing food.

"*When first thrown wholly upon a diet of reindeer meat, it seems inadequate to properly nourish the system, and there is an apparent weakness and inability to perform severe exertive fatiguing journeys. But this soon passes away in the course of two or three weeks."*

This observation, written a century before the current author first came to grips with the issue of "keto-adaptation", offers an early clue to resolve the dichotomy between impaired performance with low carbohydrate diets in the laboratory and their lack of debilitating effects when taken among people practiced in their use. That Schwatka was not impaired by his prolonged experience eating meat and fat is evidenced by his diary entry for the period 12–14 March 1880, during which he and an Inuit companion walked the last 65 miles in less than 48 hours to make a scheduled rendezvous with a whaling ship and complete his journey home.

Twenty-six years later, a Harvard-trained anthropologist named Vilhjalmur Stefansson entered the Arctic with the purpose of studying the Inuit language and culture. Having been born in 1879 in Manitoba and grown up in North Dakota, it is unlikely that Stefansson was aware of the Schwatka expedition or its reported technique of extended dogsled travel while living by hunting. However when separated from his expedition and thus his source of supply over the winter of 1906–7, Stefansson was taken in by a group of Inuit on the Canadian Arctic coast. With the arrival of spring in June of 1907, he both spoke their language and had acquired their skill of living and traveling by dogsled on a hunter's diet.

For the next decade, Stefansson traveled extensively over the arctic mainland and among the islands to the north. During this period, he was away from the outposts of European settlement for periods of up to 18 months at a time, and in the remote regions of the Canadian Arctic he lived with groups of Inuit for whom he was the first European they had met.

Stefansson wrote extensively about these experiences in both the scientific literature and in books for the lay public [7]. One of the main themes of his writing was the adaptation of the Inuit culture to survive as nomadic groups in the arctic on a diet consisting solely of the products of hunting and fishing. Coming as it did in the same time period that the science of nutrition was blossoming with the discovery and characterization of vitamins (eg, the first vitamin to be chemically defined was thiamin by Funk in 1911), Stefansson's claim that one could live and function well on the products of just one food group caused tremendous controversy [8].

Subjected to great criticism and even scorn, Stefansson agreed to recreate the Inuit diet under scientific observation. Therefore, for the calendar year of 1929 he and a colleague from his arctic explorations ate a diet consisting of meat and fat for 12 months. This experiment, supervised by Dr. Eugene DuBois, was conducted at Bellevue Hospital in New York. For the first 3 months of this study, the two explorers were under constant observation to guarantee dietary compliance, after which they were allowed more freedom of movement but with frequent tests to document that they remained in ketosis. This study was reported in multiple peer-reviewed publications, the primary reports being published in the Journal of Biological Chemistry in 1930 [9,10], As noted by DuBois [8], the study results were essentially "negative", in that both subjects survived the 12 months in apparent good health, having no signs of scurvy (which was predicted to occur within the first 3 months) or other deficiency diseases.

It is interesting to note from the careful observations published from the Bellevue study that Stafansson ate relatively modestly of protein, deriving between 80–85% of his dietary energy from fat and only 15–20% from protein [9]. This was, and still remains, at odds with the popular conception that the Inuit ate a high protein diet, whereas in reality it appears to have been a high fat diet with a moderate intake of protein. In his writings, Stefansson notes that the Inuit were careful to limit their intake of lean meat, giving excess lean meat to their dogs and reserving the higher fat portions for human consumption [11].

It is also interesting to conjecture that the vigorous defense of his arctic observations by Stefansson may have led indirectly to the development of the carbohydrate loading hypothesis. Stefansson was a polarizing influence in the field of nutrition, and his advocacy of pemmican as an emergency ration for troops during the Second World War led directly to the Kark study quoted above, which in turn was a predecessor to many comparative dietary trials performed in Europe and the U.S. in later decades.

## Modern ketogenic diet performance studies

There was a resurgence of interest in very low calorie ketogenic diets for weight loss in the 1970's, followed closely by the complications (including sudden death) associated with the Liquid Protein diet popularized in 1976. However, the fatigue and apparent cardiac dysfunction caused by this collagen-based fad diet stood in stark contrast to the published experience of arctic explorers such as Schwatka and Stefansson. In addition, physicians who monitored patients following very low calorie diets observed wide variations between the exercise-tolerance of these individuals.

Given that the elegant research on the metabolism of total fasting by Dr. George Cahill and colleagues had demonstrated that full adaptation of nitrogen, fat, and carbohydrate metabolism required a number of weeks [12], it seemed reasonable to hypothesize that exercise tolerance would take more than a week to recover after removal of carbohydrate from the diet. This view was supported by the subsequent discovery of the prescient adaptation quote from Schwatka's diary [6] noted above.

To test this hypothesis, the current author (under the mentorship of Drs. Ethan Sims and Edward Horton at the University of Vermont) undertook a study of subjects given a very low calorie ketogenic diet for 6 weeks in a metabolic research ward [13]. The protein for this diet, along with a modicum of inherent fat, was provided by lean meat, fish, and poultry providing 1.2 grams of protein per kg of reference ("ideal") body weight daily. In addition, mindful that the natriuresis of fasting could reduce circulating blood volume and cause secondary renal potassium wasting, the subjects were prescribed 3 grams of supplemental sodium as bouillion and 25 mEq (1 g) of potassium as bicarbonate daily.

Treadmill performance testing of these subjects included determinations of peak aerobic power (VO_2_max) after a 2-week weight maintenance baseline diet, and again after 6 weeks of the ketogenic weight loss diet. Endurance time to exhaustion was quantitated at 75% of the baseline VO_2_max. This endurance test was repeated again after one week of weight loss and finally after 6 weeks of weight loss. Other than these tests, the subjects did no training exercise during their participation in this study. To compensate for the fact that the average subject had lost over10 kg, the final endurance treadmill test was performed with the subject carrying a backpack equivalent in weight to the amount lost.

The energy expenditure data (expressed as oxygen consumption) and exercise times across this 8-week inpatient study are shown in Table 1. That these subjects'peak aerobic power did not decline despite 6 weeks of a carbohydrate-free, severely hypocaloric diet implies that the protein and mineral contents of the diet were adequate to preserve functional tissue. As can be noted, endurance time to exhaustion was reduced after one week of the ketogenic diet, but it was significantly increased over the baseline value by the 6-week time point. However the interpretation of this endurance test is confounded by the fact that the oxygen cost (ie, energy cost) of the treadmill exercise had significantly decreased following the weight loss, and this occurred despite the subjects being made to carry a backpack loaded to bring them back to their initial exercise test weight.

**Table 1:** Exercise parameters of Vermont study [13]

	**Baseline**	**Week 1**	**Week 6**
**VO_2_max **(LPM)	2.49	--	2.49
**Exercise VO_2_**(LPM)	1.88*	1.71	1.50*
**Endurance time **(min)	168^+^	130	249^+^

LPM, liter per minute *week 6 < baseline, P < 0.05 ^+^week 6 > baseline, P < 0.01

This question of improved efficiency notwithstanding, it is clear that our subjects experienced a delayed adaptation to the ketogenic diet, having reduced endurance performance after one week followed by a recovery to or above baseline in the period between one and six weeks. Given the reduced energy cost of the exercise despite the backpack, the extent of this adaptation cannot be determined from this study. To explain this improved exercise efficiency, we can speculate that humans are more efficient carrying weight in a modern backpack than under their skin as excess body fat. It is also possible that these untrained subjects became more comfortable with prolonged treadmill walking by their third test, and therefore improving their overall efficiency.

Given the uncertainties of this study caused by the subject's weight loss and potential for improved technique with multiple tests, the current author undertook a second study under the mentorship of Dr. Bruce Bistrian at MIT in Cambridge MA [14,15]. The diet employed in this followup study was patterned after that consumed by Stefansson during his year in the Bellevue study (and thus presumably close to that traditionally consumed by the Inuit) with the intention that the subjects would be in ketosis without weight loss.

This second study utilized competitive bicycle racers as subjects, confined to a metabolic ward for 5 weeks. In the first week, subjects ate a weight maintenance (eucaloric) diet providing 67% of non-protein energy as carbohydrate, during which time baseline performance studies were performed. This was followed by 4 weeks of a eucaloric ketogenic diet (EKD) providing 83% of energy as fat, 15% as protein, and less than 3% as carbohydrate. The meat, fish, and poultry that provided this diets protein, also provided 1.5 g/d of potassium and was prepared to contain 2 g/d of sodium. These inherent minerals were supplemented daily with an additional 1 g of potassium as bicarbonate, 3 grams of sodium as bouillon, 600 mg of calcium, 300 mg of magnesium, and a standard multivitamin.

The bicyclist subjects of this study noted a modest decline in their energy level while on training rides during the first week of the Inuit diet, after which subjective performance was reasonably restored except for their sprint capability, which remained constrained during the period of carbohydrate restriction. On average, subjects lost 0.7 kg in the first week of the EKD, after which their weight remained stable. Total body potassium (by ^40^K counting) revealed a 2% reduction in the first 2 weeks (commensurate with the muscle glycogen depletion documented by biopsy), after which it remained stable in the 4^th ^week of the EKD. These results are consistent with the observed reduction in body glycogen stores but otherwise excellent preservation of lean body mass during the EKD.

The results of physical performance testing are presented in Table 2. What is remarkable about these data is the lack of change in aerobic performance parameters across the 4-week adaptation period of the EKD. The endurance exercise test on the cycle ergometer was performed at 65% of VO_2_max, which translates in these highly trained athletes into a rate of energy expenditure of 960 kcal/hr. At this high level of energy expenditure, it is notable that the second test was performed at a mean respiratory quotient of 0.72, indicating that virtually all of the substrate for this high energy output was coming from fat. This is consistent with measures before and after exercise of muscle glycogen and blood glucose oxidation (data not shown), which revealed marked reductions in the use of these carbohydrate-derived substrates after adaptation to the EKD.

**Table 2:** Exercise parameters of MIT EKD study [15]

	**VO_2_max **(LPM)	**Exercise VO_2_**(LPM)	**Exercise RQ**	**Endurance time **(min)
**Baseline**	5.1	3.18	0.83*	147
**EKD-4**	5.0	3.21	0.72*	151

LPM, liter per minute * P < 0.01

Examining the results of these two ketogenic diet performance studies together indicates that both groups experienced a lag in performance across the first week or two of carbohydrate restriction, after which both peak aerobic power and sub-maximal (60–70% of VO_2_max) endurance performance were fully restored. In both studies, one with untrained subjects and the other with highly trained athletes who maintained their training throughout the study, there was no loss of VO_2_max despite the virtual absence of dietary carbohydrate for 4–6 weeks. This whole-body measure of oxidative metabolism could not be maintained unless there was excellent preservation of the full complement of functional tissues including skeletal muscle (and mitochondrial) mass, circulating red cell mass, and cardiopulmonary functions.

The possibility raised by the first study of improved endurance time after keto-adaptation was not substantiated by the second study employing highly trained athletes without the complicating variable of major weight loss. It is thus likely that the increased endurance time in the Vermont study was due to improved efficiency (ie, less hobbling from a backpack than from an equal weight of internal body fat) and/or improved acclimation to the endurance test procedure. Such acclimation would not be expected in the second study, as the highly trained bicycle racers were well conditioned to the stationary ergometer at the start of the study. It is also worth noting that the bicycle racers remained weight stable (excepting the half kilogram of reduced muscle glycogen) across the 4 weeks of the EKD, which was equi-caloric with the baseline diet. Although 4 weeks is a relatively short period to assess small differences in energy efficiency between diets, this observation implies that there was no great reduction in the efficiency of energy metabolism after keto-adaptation.

As a final note in this section, neither the Vermont study nor the MIT study has been refuted in the 2 decades since their publication. Understandably given the expense of human metabolic ward studies and the orthogonal conclusions of these two studies, neither study has been corroborated by a similar human study. However two subsequent animal studies examining physical performance after keto-adaptation have yielded results consistent with those presented above [16,17].

## Resolving the performance paradox

There are three factors that can help us explain the paradox presented by studies showing superior performance with high carbohydrate diets versus the present author's two studies noted above.

### Adaptation

The most obvious of these is the time allotted (or not) for keto-adaptation. In this context, the prescient observation of Schwatka (that adaptation to "a diet of reindeer meat" takes 2–3 weeks) says it all. None of the comparative low-carbohydrate versus high-carbohydrate studies done in support of the carbohydrate loading hypothesis sustained the low carbohydrate diet for more than 2 weeks [5], and most (including the classic report of Christensen and Hansen [2]) maintained their low-carbohydrate diets for 7 days or less.

There are to date no studies that carefully examine the optimum length of this keto-adapataion period, but it is clearly longer than one week and likely well advanced within 3–4 weeks. The process does not appear to happen any faster in highly trained athletes than in overweight or untrained individuals. This adaptation process also appears to require consistent adherence to carbohydrate restriction, as people who intermittently consume carbohydrates while attempting a ketogenic diet report subjectively reduced exercise tolerance.

### Sodium and potassium

The second factor differentiating the author's studies from many others is optimized mineral nutriture, which has benefits for both cardiovascular reserve in the short term and preservation of lean body mass and function over longer time periods. The Inuit people lived much of the year on coastal ice (which is partially desalinated sea water), and much of their food consisted of soup made with meat in a broth from this brackish source of water. When they went inland to hunt, they traditionally added caribou blood (also a rich source of sodium) to their soup. With these empirically derived techniques, the Inuit culture had adapted the available resources to optimize their intakes of both sodium and potassium.

When meat is baked, roasted, or broiled; or when it is boiled but the broth discarded, potassium initially present in the meat is lost, making it more difficult to maintain potassium balance in the absence of fruits and vegetables. Because our research subjects were accustomed to eating meat, fish, and poultry prepared as something other than soup, we chose to give them most of their sodium separately as bouillon and a modest additional supplement of potassium as potassium bicarbonate. With these supplements maintaining daily intakes for sodium at 3–5 g/d and total potassium at 2–3 g/d, our adult subjects were able to effectively maintain their circulatory reserve (ie, allowing vasodilatation during submaximal exercise) and effective nitrogen balance with functional tissue preservation.

An example of what happens when these mineral considerations are not heeded can be found in a study prominently published in 1980 [18]. This was a study designed to evaluate the relative value of "protein only" versus "protein plus carbohydrate" in the preservation of lean tissue during a weight loss diet. The protein only diet consisted solely of boiled turkey (taken without the broth), whereas the protein plus carbohydrate consisted of an equal number of calories provided as turkey plus grape juice. Monitored for 4 weeks in a metabolic ward, the subjects taking the protein plus carbohydrate did fairly well at maintaining lean body mass (measured by nitrogen balance), whereas those taking the protein only experienced a progressive loss of body nitrogen.

A clue to what was happening in this "Turkey Study" could be found in the potassium balance data provided in this report. Normally, nitrogen and potassium gains or losses are closely correlated, as they both are contained in lean tissue. Interestingly, the authors noted that the protein only diet subjects were losing nitrogen but gaining potassium. As noted in a rebuttal letter published soon after this report [19], this anomaly occurred because the authors assumed the potassium intake of their subjects based upon handbook values for raw turkey, not recognizing that half of this potassium was being discarded in the unconsumed broth. Deprived of this potassium (and also limited in their salt intake), these subjects were unable to benefit from the dietary protein provided and lost lean tissue. Also worthy of note, although this study was effectively refuted by a well-designed metabolic ward study published 3 years later [20], this "Turkey Study" continues to be quoted as an example of the limitations of low carbohydrate weight loss diets.

### Protein dose

The third dietary factor potentially affecting physical performance is adjusting protein intake to bring it within the optimum therapeutic window for human metabolism. The studies noted herein [13-15,20] demonstrate effective preservation of lean body mass and physical performance when protein is in the range of 1.2 – 1.7 g/kg reference body weight daily, provided in the context of adequate minerals. Picking the mid-range value of 1.5 g/kg-d, for adults with reference weights ranging from 60–80 kg, this translates into total daily protein intakes 90 to 120 g/d. This number is also consistent with the protein intake reported in the Bellevue study [9]. When expressed in the context of total daily energy expenditures of 2000–3000 kcal/d, about 15% of ones daily energy expenditure (or intake if the diet is eucaloric) needs to be provided as protein.

The effects of reducing daily protein intake to below 1.2 g/kg reference weight during a ketogenic diet include progressive loss of functional lean tissue and thus loss of physical performance, as demonstrated by Davis et al [21]. In this study, subjects given protein at 1.1 g/kg-d experienced a significant reduction in VO_2_max over a 3 month period on a ketogenic diet, whereas subjects given 1.5 g/kg-d maintained VO_2_max.

At the other end of the spectrum, higher protein intakes have the potential for negative side-effects if intake of this nutrient exceeds 25% of daily energy expenditure. One concern with higher levels of protein intake is the suppression of ketogenesis relative to an equi-caloric amount of fat (assuming that ketones are a beneficial adaptation to whole body fuel homeostasis). In addition, Stefansson describes a malady known by the Inuit as rabbit malaise [8]. This problem would occur in the early spring when very lean rabbits were the only available game, when people might be tempted to eat too much protein in the absence of an alternative source of dietary fat. The symptoms were reported to occur within a week, and included headache and lassitude. Such symptoms are not uncommon among people who casually undertake a "low carbohydrate, high protein" diet.

## Conclusions

Both observational and prospectively designed studies support the conclusion that submaximal endurance performance can be sustained despite the virtual exclusion of carbohydrate from the human diet. Clearly this result does not automatically follow the casual implementation of dietary carbohydrate restriction, however, as careful attention to time for keto-adaptation, mineral nutriture, and constraint of the daily protein dose is required. Contradictory results in the scientific literature can be explained by the lack of attention to these lessons learned (and for the most part now forgotten) by the cultures that traditionally lived by hunting. Therapeutic use of ketogenic diets should not require constraint of most forms of physical labor or recreational activity, with the one caveat that anaerobic (ie, weight lifting or sprint) performance is limited by the low muscle glycogen levels induced by a ketogenic diet, and this would strongly discourage its use under most conditions of competitive athletics.

## List of abbreviations

VO_2_max – maximum aerobic capacity

RQ – respiratory quotient

EKD – eucaloric ketogenic diet

## Competing interests

None declared.
